# Prevalence of Low Bone Mass and Osteoporosis in Ireland: the Dual‐Energy X‐Ray Absorptiometry (DXA) Health Informatics Prediction (HIP) Project

**DOI:** 10.1002/jbm4.10798

**Published:** 2023-08-10

**Authors:** John J. Carey, E Erjiang, Tingyan Wang, Lan Yang, Mary Dempsey, Attracta Brennan, Ming Yu, Wing P. Chan, Bryan Whelan, Carmel Silke, Miriam O'Sullivan, Bridie Rooney, Aoife McPartland, Gráinne O'Malley

**Affiliations:** ^1^ School of Medicine, College of Medicine, Nursing and Health Sciences University of Galway Galway Ireland; ^2^ Department of Rheumatology Galway University Hospitals Galway Ireland; ^3^ School of Management Guangxi Minzu University Nanning China; ^4^ Nuffield Department of Medicine University of Oxford Oxford UK; ^5^ Insight SFI Research Centre for Data Analytics, Data Science Institute University of Galway Galway Ireland; ^6^ School of Engineering, College of Science and Engineering University of Galway Galway Ireland; ^7^ School of Computer Science, College of Science and Engineering University of Galway Galway Ireland; ^8^ Department of Industrial Engineering Tsinghua University Beijing China; ^9^ Department of Radiology, Wan Fang Hospital Taipei Medical University New Taipei Taiwan; ^10^ Department of Rheumatology Our Lady's Hospital Manorhamilton Ireland; ^11^ Department of Geriatric Medicine Sligo University Hospital Sligo Ireland

**Keywords:** BONE MINERAL DENSITY, DXA, IRELAND, LOW BONE MASS, OSTEOPOROSIS

## Abstract

Osteoporosis is a common disease that has a significant impact on patients, healthcare systems, and society. World Health Organization (WHO) diagnostic criteria for postmenopausal women were established in 1994 to diagnose low bone mass (osteopenia) and osteoporosis using dual‐energy X‐ray absorptiometry (DXA)‐measured bone mineral density (BMD) to help understand the epidemiology of osteoporosis, and identify those at risk for fracture. These criteria may also apply to men ≥50 years, perimenopausal women, and people of different ethnicity. The DXA Health Informatics Prediction (HIP) project is an established convenience cohort of more than 36,000 patients who had a DXA scan to explore the epidemiology of osteoporosis and its management in the Republic of Ireland where the prevalence of osteoporosis remains unknown. In this article we compare the prevalence of a DXA classification low bone mass (*T*‐score < −1.0) and of osteoporosis (T‐score ≤ −2.5) among adults aged ≥40 years without major risk factors or fractures, with one or more major risk factors, and with one or more major osteoporotic fractures. A total of 33,344 subjects met our study inclusion criteria, including 28,933 (86.8%) women; 9362 had no fractures or major risk factors, 14,932 had one or more major clinical risk factors, and 9050 had one or more major osteoporotic fractures. The prevalence of low bone mass and osteoporosis increased significantly with age overall. The prevalence of low bone mass and osteoporosis was significantly greater among men and women with major osteoporotic fractures than healthy controls or those with clinical risk factors. Applying our results to the national population census figure of 5,123,536 in 2022 we estimate between 1,039,348 and 1,240,807 men and women aged ≥50 years have low bone mass, whereas between 308,474 and 498,104 have osteoporosis. These data are important for the diagnosis of osteoporosis in clinical practice, and national policy to reduce the illness burden of osteoporosis. © 2023 The Authors. *JBMR Plus* published by Wiley Periodicals LLC on behalf of American Society for Bone and Mineral Research.

## Graphical abstract

Osteoporosis prevalence in Republic of Ireland

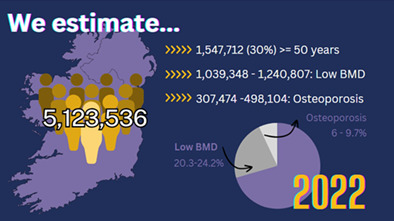

## Introduction

Osteoporosis and resulting fractures represent one of the largest disease burdens in older populations, whose fractures affect millions of patients worldwide each year.^(^ ^1^, ^2^, ^3^, ^4^, ^5^, ^6^ ^)^ The proportion of older people in Europe suffering from osteoporosis will increase substantially over the coming decade.^(^ ^4^, ^7^ ^)^ Osteoporotic fractures result in substantial morbidity, increased mortality, and an enormous economic burden.^(^ ^1^, ^2^, ^3^, ^4^, ^5^, ^6^, ^7^ ^)^ The direct cost of managing osteoporosis in Europe was almost €57 billion in 2019.^(^ ^4^ ^)^ In Sweden osteoporotic fractures are the third leading causes of death,^(^ ^4^ ^)^ whereas in the UK and USA 1‐year mortality is greater than 20% following hip and spine fractures.^(^ ^8^, ^9^ ^)^ On a global scale, osteoporotic fractures will continue to pose a serious burden for individuals and societies into the future.^(^ ^1^, ^2^, ^3^, ^4^, ^5^ ^)^

Bone mineral density (BMD) is an important surrogate for skeletal strength, and the single best predictor of fracture in older men and women without prior fracture.^(^ ^10^, ^11^, ^12^ ^)^ Measurement of BMD by dual‐energy X‐ray absorptiometry (DXA) is considered as the gold standard for noninvasive measurement and is widely available in Western Europe.^(^ ^11^, ^12^ ^)^ DXA‐measured BMD is used to identify those at risk for fracture, classify people as having “osteoporosis” or “low bone mass (osteopenia)” prior to a fracture, and monitor the effects of treatment.^(^ ^11^, ^12^, ^13^, ^14^, ^15^ ^)^ Standards for the measurement and interpretation of BMD are clear and established, in particular the use of World Health Organization (WHO) classification criteria for men ≥50 years, and perimenopausal and postmenopausal women.^(^ ^13^, ^16^, ^17^, ^18^, ^19^, ^20^ ^)^ Low bone mass (osteopenia) in these populations is defined as a DXA *T*‐score < −1.0 whereas osteoporosis may be considered when a person's DXA *T*‐score is ≤ −2.5, using the recommended reference population and in the appropriate circumstances.^(^ ^16^, ^17^, ^18^, ^19^, ^20^ ^)^ The prevalence of osteoporosis, and risk of fracture, vary considerably within and between populations, influenced by demographics, choice of test and reference population, genetics, environment, and comorbidities.^(^ ^5^, ^6^, ^20^, ^21^, ^22^, ^23^, ^24^ ^)^

Ireland has one of the largest proportional illness burdens related to osteoporosis in Europe, and projected to have the greatest increase over the coming decade.^(^ ^4^ ^)^ The cost of managing those who fracture will double by 2030.^(^ ^25^ ^)^ Despite some progress in understanding fracture admissions to public hospitals among older persons,^(^ ^26^, ^27^, ^28^ ^)^ national validated data do not exist related to the prevalence of osteoporosis or incidence of major osteoporotic fracture.^(^ ^27^, ^28^, ^29^, ^30^, ^31^ ^)^ This may be contributing to why osteoporosis is not a national healthcare priority.^(^ ^4^ ^)^ We established a large convenience cohort of >36,000 patients across multiple sites to examine the epidemiology of osteoporosis in Ireland, and to assess the validity of DXA biometrics, algorithms, and classification criteria for Irish adults.^(^ ^30^ ^)^ We have previously shown that the prevalence of vertebral fractures increases with age in older men and women,^(^ ^31^, ^32^ ^)^ and that the international recommendation to use the Third National Health and Nutrition Examination Survey (NHANES III) white female reference data appears appropriate for our population.^(^ ^33^ ^)^ In this work we compare the BMD and prevalence of low bone mass and osteoporosis among men and women referred for a DXA scan, between those without major risk factors or fracture, to those with major risk factors but without fractures, and those with major osteoporotic fractures.

## Patients and Methods

### Data source

Data were obtained from four GE Lunar DXA machines (GE Lunar, Madison, WI, USA) in three hospitals in western Ireland following approval by the hospitals ethics committee. All DXA staff are trained and certified to the standards recommended by the International Society for Clinical Densitometry (ISCD).^[^ ^13^ ^]^ Data on >36,000 patients scanned between January 2000 and November 2018 were extracted, cleaned, anonymised, and merged in compliance with European Union General Data Protection Regulation (GDPR) legislation.^(^ ^34^ ^)^ Details of the data extraction, cleaning and analytic processes, and of the cohort, including demographics, risk factors, and treatments have been described.^(^ ^30^, ^31^, ^32^, ^33^, ^35^ ^)^ We used NHANES III white female reference data to calculate *T*‐scores at the femoral neck and total hip, and manufacturer USA/Northern Europe (GE Lunar) white female reference data to calculate lumbar spine and 1/3 radius *T*‐scores. We chose the lowest *T*‐score at either the lumbar spine, femoral neck, or total hip for diagnosis, as recommended by the ISCID.^(^ ^12^, ^13^, ^17^, ^18^, ^19^ ^)^ Detailed 2016 National Census Population Statistics were used to estimate the prevalence of low bone mass and osteoporosis across each decade and gender among those aged ≥50 years, and preliminary data from 2022, to estimate the prevalence for our population.^(^ ^36^ ^)^

### Study eligibility criteria

**Fig. 1:**
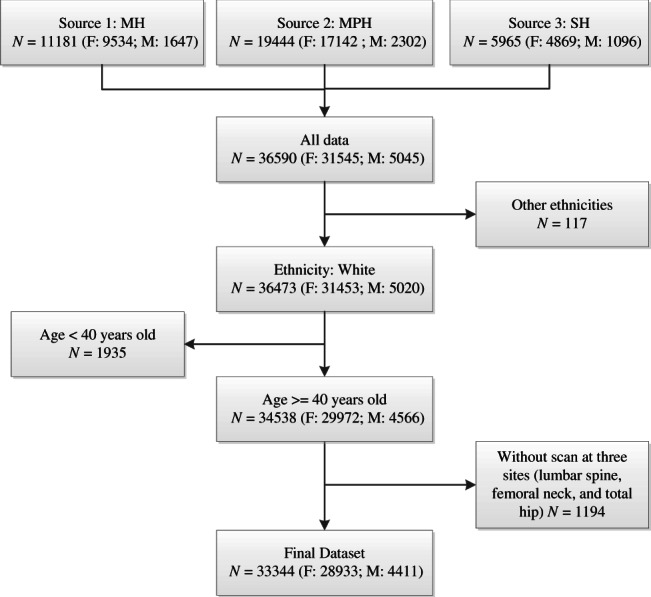
Flow diagram of the subject selection process.

In this study, we included all patients aged ≥40 years and older who had suitable DXA scan data for analysis (Fig. 1). For patients with more than one scan, only their baseline scan was included in this study. We excluded adults aged <40 years (1935), non‐whites (117), and adults without a scan at the spine or hip (1194).

We chose to compare the mean BMD, and prevalence of low bone mass (*T*‐score < −1.0) and osteoporosis (*T*‐score ≤ −2.5) between men and women without fractures or major osteoporotic risk factors (“None”), those with major risk factors for fracture but without prior fractures (“Risk”), and those with a prior major osteoporotic fractures (“Fracture”).

### Statistical analysis methods

We summarized the number, height, weight, body mass index (BMI), and BMD for measured skeletal sites as recommended by ISCD, in total and self‐defined gender. Risk factors were considered as binary variables for the purposes of this study as outlined.^(^ ^30^ ^)^ We further subdivided those with risk factors into subgroups of people with one, two, three, or four or more risk factors. We used the Student two‐sample *t* test, analysis of variance (ANOVA), and analysis of covariance (ANCOVA) methods to compare mean differences between groups, and two‐sample χ^2^ tests to compare proportions. All statistical analyses were conducted with R Studio for Windows (Version 3.5.1; https://github.com/rstudio/rstudio).

## Results

### Characteristics of study subjects

Data were available for a total of 33,344 (91%) men and women who met our study inclusion criteria, outlined in Fig. 1 and Table 1. The majority were women (86.8%) with a mean age >62 years and BMI of 26.7. More than 95% of subjects had a suitable hip scan available, 83% a suitable spine scan, whereas 26,487 (79.4%) had a DXA scan of both the spine and hip available (Table 1). Only 5% of subjects had a forearm scan so we have not included those results in this study. The number of adults aged >90 years (*n* = 164) was small. Men were generally older, taller, and heavier than women (Table 1). Although age was similar for men across the three cohorts (*p* = 0.629), the age of women was significantly different (*p* < 0.001), being lowest for those with risk factors, and greatest for those with fractures (Table 2). The most common reason for DXA referral was a prior fracture, but other major risk factors such as family history of osteoporosis, corticosteroid therapy, inflammatory arthritis, cancer therapy, and tobacco use^(^ ^30^, ^37^ ^)^ were common. Table 2 shows that 9362 (28%) subjects did not have a major risk factor for, or prior, fracture, including 985 men and 8377 women other than age; 9050 subjects had at least one fracture, at sites categorized as major osteoporotic fractures by the International Osteoporosis Foundation.^(^ ^7^ ^)^ Fractures present on vertebral fracture assessment (VFA) scans were not included in the data available to use for this study.

**Table 1:** Brief Descriptive Summary of Our Study Population

Parameter	Total *N* = 33,344 (100%)	Women *n* = 28,933 (86.8%)	Men *n* = 4411 (13.2%)
Age in years, mean ± SD	63.01 ± 11.20	62.52 ± 11.04^a^	66.22 ± 11.68
Weight in kg, mean ± SD	70.25 ± 14.69	68.56 ± 13.81^a^	81.34 ± 15.47
Height in cm, mean ± SD	161.83 ± 7.74	160.25 ± 6.50^a^	172.18 ± 7.20
BMI kg/m^2^, mean ± SD	26.78 ± 5.10	26.69 ± 5.15^a^	27.38 ± 4.67
Number of scans, mean ± SD	1.46 ± 0.89	1.48 ± 0.92	1.29 ± 0.69
Lumbar spine, *n* (%)	27,732 (83.2)	24,416 (84.4)	3316 (75.2)
Femoral neck, *n* (%)	32,138 (96.4)	27,931 (96.5)	4207 (95.4)
Right site, *n* (%)	23,984 (71.9)	20,363 (70.4)	3621 (82.1)
Left site, *n* (%)	19,264 (57.8)	16,780 (58.0)	2484 (56.3)
Total hip, *n* (%)	32,171 (96.5)	27,957 (96.6)	4214 (95.5)
Right site, *n* (%)	24,184 (72.5)	20,554 (71.0)	3630 (82.3)
Left site, *n* (%)	19,303 (57.9)	16,811 (58.1)	2.492 (56.5)
All three sites, *n* (%)	26,487 (79.4)	23,373 (80.8)	3114 (70.6)

*Note*: The Student *t* test was used to compare the mean age, weight, height, and BMI of female cohort and male cohort.

^a^ *p* < 0.001.

**Table 2:** Comparison of Mean Bone Mineral Density (g/cm^2^) at Each Skeletal Site of Men and Women Between Healthy Control Population (None), Those at Risk, and Those With Fractures

	None (*n* = 9362)		Risk factor (*n* = 14,932)		Fracture (*n* = 9050)	
Group	Number	Mean ± SD	Number	Mean ± SD	Number	Mean ± SD
Women, *n*	8377		12,704		7852	
Age (years)		62.31 ± 10.98		60.84 ± 10.57^a^		65.45 ± 11.26^a^
Lumbar spine	7274	1.058 ± 0.186	10,837	1.057 ± 0.181^a^	6305	0.997 ± 0.182^a^
Femoral neck	8094	0.855 ± 0.144	12,308	0.858 ± 0.140^a^	7529	0.797 ± 0.137^a^
Total hip	8093	0.903 ± 0.156	12,304	0.907 ± 0.155^a^	7560	0.839 ± 0.155^a^
Men, *n*	985		2228		1198	
Age (years)		65.97 ± 12.71		66.20 ± 11.23		66.45 ± 11.60
Lumbar spine	807	1.175 ± 0.228	1659	1.173 ± 0.214	850	1.098 ± 0.217
Femoral neck	917	0.902 ± 0.170	2144	0.903 ± 0.160	1146	0.846 ± 0.149
Total hip	918	0.980 ± 0.177	2149	0.979 ± 0.169	1147	0.907 ± 0.170

*Note*: ANOVA analysis compares age of three subgroups in female cohort (*p* < 0.001) and male cohort (*p* = 0.629). ANCOVA analysis compares the BMD of three subgroups in female and male, respectively, adjusting for the variables of age, group, and interactions between age and group.

^a^ *p* < 0.001.

### Risk factors

A total of 14,932 subjects had one or more major risk factor for fracture, but no prior fracture, including 2228 (15%) men and 12,704 (85%) women. The majority (9064, 61%) had only one major risk factor identified (Table 3), whereas the average number of risk factors per person was 1.9. The most frequently noted risk factors were a family history of osteoporosis (22.3%), tobacco use (14.3%), corticosteroid use (18.8%), inflammatory arthritis (9.9%), alcohol excess (0.7%), hypogonadism and hormonal therapy for cancer (29.1%), and other (46.2%).

**Table 3:** Comparison of Bone Mineral Density Between Men and Women Without Major Risk Factors or Fracture to Those With Risk Factors But Without Fractures

	None (*n* = 9362)		One (*n* = 9064)		Two (*n* = 4195)		Three (*n* = 1312)		Four or more (*n* = 361)	
Number of risk factors	*n*	Mean ± SD	*n*	Mean ± SD	*n*	Mean ± SD	*n*	Mean ± SD	*n*	Mean ± SD
Women	8377		7785		3493		1102		324	
Lumbar spine	7274	1.058 ± 0.186	6752	1.058 ± 0.186	2930	1.056 ± 0.184	893	1.050 ± 0.184	262	1.055 ± 0.187
Femoral neck	8094	0.855 ± 0.144	7531	0.860 ± 0.139^c^	3387	0.856 ± 0.139	1070	0.853 ± 0.147	320	0.846 ± 0.144
Total hip	8093	0.903 ± 0.156	7512	0.910 ± 0.154^b^	3399	0.904 ± 0.155	1073	0.901 ± 0.162	320	0.891 ± 0.163
Men	985		1279		702		210		37	
Lumbar spine	807	1.175 ± 0.228	977	1.178 ± 0.218	498	1.165 ± 0.203^b^	158	1.166 ± 0.218^a^	26	1.173 ± 0.266^a^
Femoral neck	917	0.902 ± 0.170	1230	0.907 ± 0.156	679	0.898 ± 0.164	199	0.894 ± 0.166	36	0.899 ± 0.170
Total hip	918	0.980 ± 0.177	1233	0.985 ± 0.163	680	0.974 ± 0.175	200	0.965 ± 0.182	36	0.984 ± 0.179

*Note*: Differences between risk‐free patients and one or more risk patients are derived from one‐factor ANOVA.

^a^ *p* < 0.001.

^b^ *p* < 0.01.

^c^ *p* < 0.05.

### Fractures

A total of 9500 subjects were noted to have at least one major osteoporotic fracture, including 1198 (13%) men and 7852 (86.8%) women (Table 2). These include 3140 with forearm and wrist fractures, 877 with vertebral fractures, 784 with hip fractures, 635 with humeral fractures, and 2214 with osteoporotic fractures at other sites. The majority (6807/9050, 75%) of those with prevalent fractures had at least one additional major risk factor for fracture (Table 4): family history of osteoporosis (13.9%), tobacco use (11.5%), corticosteroid use (10.0%), inflammatory arthritis (6.2%), alcohol excess (1.2%), hypogonadism and hormonal therapy for cancer (15.3%), and other (31.3%). We were unable to verify the site and nature of prior fracture among 2841 (30.7%) of patients. Additional analyses after excluding these subjects with a “possible” prior fracture are included in another work, which did not have a major impact on the overall results in terms of age, BMI or BMD (Author and colleagues, unpublished data).

**Table 4:** Mean Bone Mineral Density of Men and Women With Fractures With and Without Additional Risk Factors

	Fracture without risk factors (*n* = 2243)		Fracture with risk factors (*n* = 6807)	
Group	*n*	Mean ± SD	*n*	Mean ± SD
Women	1933		5919	
Age		66.11 ± 11.78		65.23 ± 11.08
Lumbar spine	1539	1.002 ± 0.180	4766	0.996 ± 0.183
Femoral neck	1848	0.798 ± 0.137	5681	0.797 ± 0.137
Total hip	1857	0.838 ± 0.154	5703	0.839 ± 0.156
Men	310		888	
Age		67.14 ± 11.91		66.21 ± 11.48
Lumbar spine	217	1.101 ± 0.222	633	1.098 ± 0.215
Femoral neck	292	0.853 ± 0.157	854	0.844 ± 0.146
Total hip	292	0.914 ± 0.179	855	0.904 ± 0.167

*Note*: The Student *t* test was used to compare the mean BMD of female cohort and male cohort. All *p* values were > 0.05 (not statistically significant).

### BMD in different risk factor groups

Men had higher BMD than women (Table 2) at all measured sites, across the three distinct cohorts (none, risk factor, and fracture). BMD decreased with age in both men and women such that the prevalence of low bone mass and osteoporosis was greater among older adults in all three cohorts and both genders (Table 5). This trend was particularly striking for women (Table 5), among whom the prevalence increased several fold between from 40 to 49 years to those ≥80 years.

**Table 5:** Prevalence of Low Bone Mass (*T*‐score < −1.0) and Osteoporosis (*T*‐score ≤ −2.5) in Men and Women Among the Three Distinct Cohorts

	None			Risk factor			Fracture		
Age Category by Gender	*n*	Low bone mass *n* (%)	Osteoporosis *n* (%)	*n*	Low bone mass *n* (%)	Osteoporosis *n* (%)	*n*	Low bone mass *n* (%)	Osteoporosis *n* (%)
Women									
40–49 years	1055	442 (41.9)	47 (4.45)	1,949	979 (50.23)^a^	122 (6.26)^c^	649	383 (59.01)^a^	77 (11.86)^a^
50–59 years	2756	1588 (57.62)	360 (13.06)	4476	2762 (61.71)^a^	681 (15.21)^c^	2,076	1527 (73.55)^a^	440 (21.19)^a^
60–69 years	2438	1828 (74.98)	599 (24.57)	3639	2726 (74.91)	944 (25.94)	2,308	1929 (83.58)^a^	740 (32.06)^a^
70–79 years	1527	1315 (86.12)	592 (38.77)	2019	1721 (85.24)	745 (36.90)	1,916	1717 (89.61)^a^	862 (44.99)^a^
80–89 years	566	525 (92.76)	314 (55.48)	597	555 (92.96)	310 (51.93)	828	796 (96.14)^c^	519 (62.68)^a^
90+ years	35	34 (97.14)	24 (68.57)	24	24 (100)	12 (50.00)	75	75 (100)	58 (77.33)^c^
Total	8377	5732 (68.43)	1,936 (23.11)	12,704	8,767 (69.00)	2,814 (22.15)	7,852	6427 (81.85)^a^	2,696 (34.34)^a^
Men									
40–49 years	131	60 (45.8)	16 (12.21)	212	127 (59.91)^c^	33 (15.57)	106	87 (82.08)^a^	30 (28.3)^c^
50–59 years	188	104 (55.32)	21 (11.17)	432	280 (64.81)^c^	59 (13.66)	258	191 (74.03)^c^	60 (23.26)^b^
60–69 years	253	157 (62.06)	38 (15.02)	682	424 (62.17)	105 (15.40)	330	249 (75.45)^a^	92 (27.88)^a^
70–79 years	265	181 (68.3)	49 (18.49)	679	469 (69.07)	138 (20.32)	342	290 (84.8)^a^	123 (35.96)^a^
80–89 years	138	102 (73.91)	66 (47.83)	214	162 (75.7)	55 (25.70)^a^	151	134 (88.74)^b^	83 (54.97)^a^
90+ years	10	9 (90.00)	5 (50.00)	9	9 (100)	3 (33.33)	11	9 (81.82)	7 (63.64)
Total	985	613 (62.23)	195 (19.80)	2,228	1,471 (66.02)^c^	393 (17.64)	1,198	960 (80.13)^a^	395 (32.97)^a^

*Note*: The *χ* ^2^ test were used for the prevalence of low bone mass and osteoporosis of subgroups in female cohort and male cohort (None versus Risk Factor, Risk Factor versus Fracture).

^a^ *p* < 0.001.

^b^ *p* < 0.01.

^c^ *p* < 0.05.

**Fig. 2:**
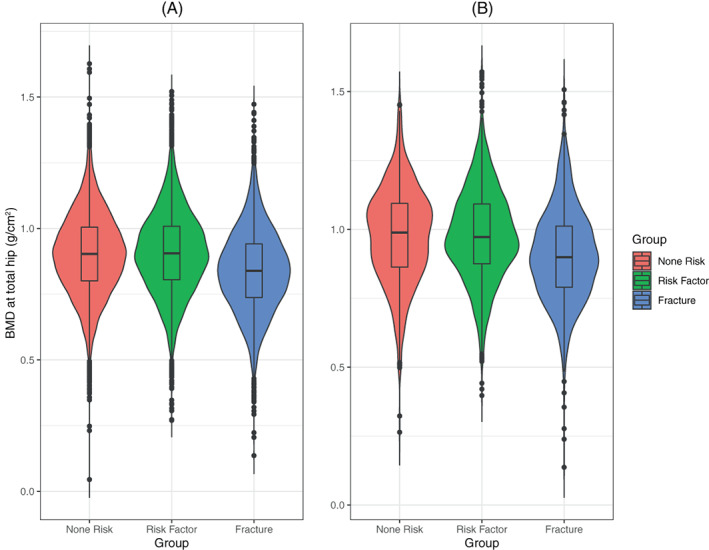
BMD at total hip for patients without fractures or major osteoporotic risk factors (“None”), those with major risk factors for fracture but without prior fractures (“Risk”), and those with a prior major osteoporotic fractures (“Fracture”) in (*A*) females and (*B*) males. BMD = bone mineral density.

Mean BMD of men and women with and without risk factors was significantly greater than that of men and women with prevalent fractures at all measured sites (Table 2, Fig. 2*A*,*B*). We found no significant trend in BMD when we compared BMD at each skeletal site and the number of additional risk factors (Table 3) for both men and women. There was no significant overall trend even comparing men and women with four or more risk factors to those with none. In addition we found no significant difference between mean BMD of both men and women with a prior major osteoporotic fracture who had additional risk factors and those who did not. However, the number of men with fractures and no risk factors was very small (Table 4).

The majority of subjects (71.9%) had low bone mass and one in four (25.3%) had osteoporosis using DXA *T*‐score classification. The prevalence of low bone mass and osteoporosis increases with advancing age, from 50.7% and 7.9% for adults aged 40–49 years, rising to 97.6% and 66.5% among those aged ≥90 years, respectively. The overall prevalence of low bone mass and osteoporosis in the healthy cohorts was lower than those with risk factors or prevalence major osteoporotic fractures, as detailed in Table 5. The overall prevalence among those with fractures was significantly greater at 81.6% and 34.2%, respectively, compared to the healthy cohort or the risk factor cohort, *p* < 0.01, whereas <10% of fracture subjects had a *T*‐score ≤ −2.5 at all measured sites. The overall prevalence of low bone mass and osteoporosis among those with prevalent fractures was similar between men and women, in comparison to the other cohorts (Table 5).

The Irish population was 4,761,865 in 2016, almost 1.5 million of whom were aged ≥50 years, and preliminary 2022 census results show the overall population to be 5,123,536, an increase of 7.6%.^(^ ^36^ ^)^ Using these detailed data from the 2016 census a correction factor of 7% for 2022, we estimate between 1,039,348 and 1,240,807 Irish men and women aged ≥50 years have low bone mass and between 308,474 and 498,104 men and women aged ≥50 years have osteoporosis (Table 6). These numbers reflect proportions of 20.3% and 24.%, and 6.0% and 9.7% of the total population, respectively. We also examined the prevalence of low bone mass and osteoporosis among subjects during different time periods and found a greater prevalence among those scanned at an earlier date, as shown in Table 7.

**Table 6:** Estimated Prevalence of Low Bone Mass and Osteoporosis Using 2016 Census Data For Ireland

Age‐group (years)	Men		Women		Total	
	Healthy	Fracture	Healthy	Fracture	Healthy	Fracture
Population aged 50–59 years, *n*	282,070		287,967		570,037	
With low bone mass, *n*	156,041	208,816	165,927	211,800	321,968	420,616
With osteoporosis, *n*	31,507	65,722	37,608	63,065	69,115	128,787
Population aged 60–69 years, *n*	223,659		226,433		450,092	
With low bone mass, *n*	138,803	168,751	169,621	189,253	308,424	358,004
With osteoporosis, *n*	33,594	62,401	55,408	72,685	89,002	135,086
Population aged 70–79 years, *n*	133,618		144,121		277,739	
With low bone mass, *n*	91,261	113,308	122,849	129,150	214,110	242,458
With osteoporosis, *n*	24,706	48,102	45,331	64,854	70,037	112,956
Population Aged ≥80 years, *n*	58,258		90,334		148,592	
With low bone mass, *n*	43,053	51,698	83,794	86,857	126,847	138,555
With osteoporosis, *n*	14,972	32,042	45,167	56,646	60,139	88,688
Total population aged ≥50 years in 2016, *n* ^a^	697,605		748,855		1,446,460	
With low bone mass in 2016, *n*	429,163	542,573	542,191	617,060	971,354	1,159,633
With osteoporosis in 2016, *n*	104,779	208,267	183,514	257,250	288,293	465,517
Total population aged ≥50 years in 2022, *n* ^b^	746,437		801,275		1,547,712	
With low bone mass in 2022, *n*	459,204	580,553	580,144	660,254	1,039,348	1,240,807
With osteoporosis in 2022, *n*	112,114	222,846	196,360	275,258	308,474	498,104

^a^ Irish Population Census Data for 2016; www.cso.ie.

^b^ Irish Population Preliminary Census Data for 2022 suggest 7% increase: 2016 numbers multiplied by 1.07.

**Table 7:** Sensitivity Analysis of Prevalence of Low Bone Mass and Osteoporosis

Time Period DXA scan Perfomed	None			Risk factor			Fracture		
	*n*	Low bone mass *n* (%)	Osteoporosis *n* (%)	*n*	Low bone mass *n* (%)	Osteoporosis *n* (%)	*n*	Low bone mass *n* (%)	Osteoporosis *n* (%)
Women									
Total	8377	5732 (68.43)	1936 (23.11)	12,704	8767 (69.00)	2814 (22.15)	7852	6427 (81.85)	2696 (34.34)
G1:2000–2005	2690	2112 (78.51)	855 (31.78)	3975	3187 (80.18)	1191 (29.96)	1958	1707 (87.18)	833 (42.54)
G2:2006–2010	3357	2107 (62.76)^a^	623 (18.56)^a^	5164	3263 (63.19)^a^	934 (18.09)^a^	3293	2626 (79.74)^a^	1079 (32.77)^a^
G3:2011–2018	2330	1513 (64.94)	458 (19.66)	3565	2317 (64.99)	689 (19.33)	2601	2094 (80.51)	784 (30.14)
Men									
Total	985	613 (62.23)	195 (19.80)	2228	1471 (66.02)	393 (17.64)	1198	960 (80.13)	395 (32.97)
G1:2000–2005	224	138 (61.61)	46 (20.54)	237	170 (71.73)	51 (21.52)	108	89 (82.41)	42 (38.89)
G2:2006–2010	335	220 (65.67)	78 (23.28)	758	520 (68.60)	127 (16.75)	495	396 (80.00)	169 (34.14)
G3:2011–2018	426	255 (59.86)	71 (16.67)^b^	1233	781 (63.34)^b^	215 (17.44)	595	475 (79.83)	184 (30.92)

*Note*: The χ^2^ test was used for the prevalence of low bone mass and osteoporosis of subgroups in female cohort and male cohort (G1 vs. G2, G2 vs. G3).

^a^ *p* < 0.001.

^b^ *p* < 0.05.

## Discussion

Herein we describe for the first time a reasonable estimate of the true prevalence of low bone mass and osteoporosis in a large cohort of older Irish adults using Irish data, including those with and without major risk factors for osteoporosis, and those with and without prevalent major osteoporotic fractures. The prevalence of a DXA diagnosis of low bone mass and osteoporosis increased with age across both genders and each cohort, more notably among women. More than one‐half of the men and women without fractures had low bone mass (*T*‐score < −1.0) at one or more skeletal sites, whereas almost one in five had osteoporosis (*T*‐score ≤ −2.5) at one or more skeletal sites. The majority of those with a previous major osteoporotic fracture had low bone mass, whereas only one in three had a *T*‐score ≤ −2.5. We estimate that in 2022 more than 1 million Irish adults aged ≥50 years have low bone mass, whereas more than one quarter million (>250,000) have osteoporosis. These results are much higher than those contained in a recent European report,^(^ ^4^ ^)^ which have important implications for studies estimating the prevalence of osteoporosis in older men and women in Ireland, and use of DXA testing for the diagnosis of osteoporosis among those with, and at risk for, major osteoporotic fractures.

The prevalence of low bone mass increases as people age, in both men and women, and different ethnicities.^(^ ^22^, ^24^, ^38^ ^)^ This occurs in parallel with the greater incidence of fragility fractures, particularly those with the greatest morbidity, mortality, and healthcare costs; ie, hip and spine.^(^ ^1^, ^2^, ^7^, ^39^, ^40^ ^)^ Because there is significant overlap between those who will and will not fracture, no single threshold can identify such people with certainty.^(^ ^41^, ^42^ ^)^ Combining BMD with age, gender, and other risk factors greatly enhances fracture risk prediction, which increases with the number of additional factors.^(^ ^21^, ^39^, ^40^, ^43^ ^)^ Their mechanism is somewhat, though not entirely, independent of effects on BMD.^(^ ^21^, ^39^, ^43^ ^)^ Interestingly in our study, there was no “dose” effect of risk factors on the level of low bone mass, which contrasts with fracture risk prediction. The lower prevalence among subjects scanned at a later date could reflect greater osteoporosis awareness and referral patterns, population health, or other factors.

In 1994 a WHO multinational group of experts published a report that established diagnostic criteria based on measurement of BMD or bone mineral content (BMC) at the spine, forearm, or proximal femur to identify postmenopausal women at risk for, and with osteoporosis.^(^ ^16^ ^)^ The authors noted several caveats, and acknowledge some of the limitations and uncertainties.^(^ ^16^ ^)^ Some were addressed in a later publication,^(^ ^44^ ^)^ or were clarified and modified by other organizations.^(^ ^13^, ^17^ ^)^ However, the diagnosis remains dependent in part on the clinical context, population demographics, normative reference data, skeletal site, quality of the scan and report, and technology used.^(^ ^12^, ^13^, ^14^, ^23^, ^24^, ^40^, ^45^ ^)^ Large studies show these criteria have limited sensitivity because the majority of fractures occur among adults, whose fractures do not meet the DXA threshold for a diagnosis of osteoporosis.^(^ ^12^, ^42^, ^46^ ^)^ Similarly, in this study the majority of men and women known to have previous major fragility fracture did not meet the DXA threshold for osteoporosis. This was particularly striking in younger adults where only one in five women and one in four men aged 40–59 years had a *T*‐score ≤ −2.5 at any site. Similarly, a diagnosis of osteoporosis should not be made in younger men or premenopausal women on the basis of densitometric criteria alone, and it is very likely there are a considerable number of “false positive” tests in those aged 40–49 years, particularly those without major risk factors or fractures.^(^ ^13^, ^18^ ^)^ Despite the limitations, DXA measured BMD remains an appropriate choice for clinicians to diagnose osteoporosis in perimenopausal or postmenopausal women, or men aged ≥50 years without a major osteoporotic fracture in the appropriate clinical context.^(^ ^11^, ^13^ ^)^

A number of robust, costly, and time‐consuming studies have established “normative” references using carefully selected representative populations, a complex process and weighty task.^(^ ^22^, ^24^, ^38^, ^45^ ^)^ Data such as these have been used to estimate the prevalence of osteoporosis, suggesting low bone mass and osteoporosis ranges between 37% and 50% and 7.9% and 18% in postmenopausal women, whereas in men aged ≥50 years they range from 15% and 33% and 1% and 6.6%, respectively.^(^ ^23^, ^24^, ^43^ ^)^ The ISCD and International Osteoporosis Foundation currently recommend using the NHANES III proximal femur white female reference as the appropriate universal reference to calculate *T*‐scores for the diagnosis of osteoporosis in older men and postmenopausal women using WHO criteria,^(^ ^13^, ^20^ ^)^ which show a similar age‐related pattern of proximal femur BMD to our population.^(^ ^33^ ^)^ In 2016 our National Census demonstrated 1,446,460 Irish men and women aged ≥50 years, representing 30.4% of the population, though only preliminary data are currently available for 2022.^(^ ^36^ ^)^ Based on these numbers, and our results, we estimate more than 1 million have low bone mass, and more than 300,000 have osteoporosis by DXA classification criteria. We believe this is a reasonable estimate because the population has increased by more than 7% in 2022,^(^ ^36^ ^)^ we have not accounted for those aged <50 years, and we made estimates using the lowest and greatest proportions of all three cohorts to estimate these data.

Our study has several important strengths and limitations, which need consideration. We have a large population, similar in size to the original Fracture Risk Assessment Tool (FRAX) multicohort population,^(^ ^40^ ^)^ and larger than the Canadian Multicentre Osteoporosis Study (CaMos), NHANES, and Korea National Health and Nutrition Examination Survey (KNHANES) populations.^(^ ^22^, ^24^, ^38^, ^43^ ^)^ However, our population represents patients referred for a DXA examination by a medical practitioner, were only white, and were only scanned in the West of Ireland, which may not be an accurate reflection of the rest of population, and there is considerable imbalance between the proportion of subjects available for each gender and age category. We categorized people into “healthy,” at risk, and with prevalent fractures; we are able to get a clear and consistent picture of the pattern of BMD according to age, gender, and risk status. Additional centers and regions for future iterations would be welcome. Larger studies include those with prior fractures and other risk factors,^(^ ^21^, ^22^, ^38^, ^39^, ^43^ ^)^ and cohorts differ,^(^ ^24^, ^39^, ^40^, ^47^ ^)^ not too dissimilar to our population, so our use of these data are not inappropriate to assess data applicable to our local population, in particular people who are referred for a DXA scan. Although these reports show many subjects had prior fractures, or other risk factors for fracture,^(^ ^22^, ^23^, ^24^, ^38^ ^)^ and mean BMD values are broken down by decade, gender, and ethnicity,^(^ ^22^, ^24^, ^38^ ^)^ this is the first article we are aware of to publish the data segregated out by those deemed low risk (none), at risk, or with fractures. We have limited data to validate all fractures, medications, and clinical risk factors for every single patient, because there is no national electronic medical record and had no dedicated research time heretofore. Trained experienced nurses, radiographers, and clinicians collect and validate patient data at the time of scanning or reporting; it is more likely than not there are some missing or incorrect data on a minority of patients. Some imaging studies of fractures were performed in other locations and whose results are not accessible; fractures on VFA scans were not available for these analyses. Prior studies show fractures, particularly vertebral fractures, are underreported in Ireland.^(^ ^27^, ^28^, ^29^, ^32^ ^)^ We have not performed analyses of those with and without these validated fractures, which could affect our results. Risk factors and fractures are categorized simply as binary variables, rather than weighted based on a dose, duration, or severity. This is likely an important aspect for some items such as glucocorticoids, rheumatoid arthritis, and prior fracture,^(^ ^21^, ^39^ ^)^ and warrants consideration for future projects. Although a minority of women aged 40 to 49 years (25%) and 50 to 59 years (2%) were noted to be premenopausal, these DXA criteria should be applied to such populations. Finally, we have not included additional analyses with the results of forearm scans.

In conclusion, we have shown for the first time in a large Irish population that low bone mass defined by DXA classification is common in men and women aged ≥50 years whether they have risk factors or not, or previous osteoporotic fractures. The presence of osteoporosis increases with age, and is significantly greater among men and women with a prior major osteoporotic fracture. These data suggest the prevalence of osteoporosis and low bone mass is much greater than was previously suggested,^(^ ^4^ ^)^ reflecting an urgent need to establish a national programme in line with other common noncommunicable diseases. Future projects will assess the validity of various fracture risk tools for our population, and collaboration with other centers to add robustness and validity to future studies.

## Author Contributions

**John J. Carey:** Conceptualization; data curation; methodology; writing – original draft; writing – review and editing. **Erjiang E:** Formal analysis; methodology; writing – original draft; writing – review and editing. **Tingyan Wang:** Formal analysis; writing – review and editing. **Lan Yang:** Formal analysis; writing – review and editing. **Mary Dempsey:** Conceptualization; writing – review and editing. **Attracta Brennan:** Writing – review and editing. **Ming Yu:** Conceptualization. **Wing P. Chan:** Conceptualization. **Bryan Whelan:** Data curation; writing – review and editing. **Carmel Silke:** Data curation. **Miriam O'Sullivan:** Data curation. **Bridie Rooney:** Data curation. **Aoife McPartland:** Data curation. **Gráinne O'Malley:** Data curation.

## Disclosures

All authors declare they have no conflicts of interest.

### Peer Review

The peer review history for this article is available at https://www.webofscience.com/api/gateway/wos/peer-review/10.1002/jbm4.10798.

## Data Availability

The datasets analyzed during the current study are not publicly available to protect confidentiality and privacy of participants, but may be made available from the corresponding author on reasonable request.
